# Whole Lung Irradiation in Adults with Metastatic Ewing Sarcoma: Practice Patterns and Implications for Treatment

**DOI:** 10.1155/2015/591698

**Published:** 2015-06-25

**Authors:** Shyam K. Tanguturi, Suzanne George, Karen J. Marcus, George D. Demetri, Elizabeth H. Baldini

**Affiliations:** ^1^Harvard Radiation Oncology Program, Boston, MA 02115, USA; ^2^Center for Sarcoma and Bone Oncology, Dana-Farber Cancer Institute, Boston, MA 02115, USA; ^3^Department of Medical Oncology, Dana-Farber Cancer Institute, Boston, MA 02115, USA; ^4^Department of Radiation Oncology, Dana-Farber Cancer Institute, Boston, MA 02115, USA; ^5^Department of Radiation Oncology, Brigham and Women's Hospital, Boston, MA 02115, USA; ^6^Department of Radiation Oncology, Boston's Children's Hospital, Boston, MA 02115, USA

## Abstract

*Background*. Whole lung irradiation (WLI) is a standard treatment component for children with metastatic Ewing Sarcoma (ES), but data on WLI for adults are sparse. *Design*. An email survey was sent to expert sarcoma-dedicated oncologists worldwide: *An adult with excellent performance status presents with primary ES in the leg and multiple pulmonary metastases. The patient achieves complete radiographic response after chemotherapy and resection of the primary. Would you give bilateral WLI to (1) this adult patient?, (2) this patient if 20 years old (yo)?, (3) this patient if 45 yo?, or (4) this patient if 60 yo? Results*. 38 experts responded, including 24 adult, 1 adolescent young adult, and 13 pediatric oncologists. 63%, 63%, 62%, and 50% of respondents offered WLI to the adult, 20-year-old, 45-year-old, and 60-year-old, respectively. Pediatric oncologists more likely endorsed WLI across all ages including the adult (*P* = 0.01), 20-year-old (*P* = 0.005), 45-year-old (*P* = 0.01), and 60-year-old (*P* = 0.08). There were no significant differences between medical and radiation oncologists or between European/Australian and American providers. *Conclusions*. Almost two-thirds of experts surveyed supported WLI for adults with metastatic ES up to age 45 and half supported WLI for a 60-year-old. Continued collaboration across adult and pediatric oncology is needed to define evidence-based strategies across the age spectrum.

## 1. Introduction

Ewing Sarcoma (ES) is a mesenchymal malignancy of unclear histogenetic derivation characterized by distinct chromosomal translocations at the EWSR1 gene [1]; this disease occurs primarily in children and young adults and less commonly in older adults. Although 20–25% of patients with ES present with distant metastatic disease at diagnosis [2], some patients, such as those with limited pulmonary metastases (PM), may be cured with aggressive multimodality therapy. Whole lung irradiation (WLI) is one such treatment designed with curative-intent for patients with PM. In the Intergroup Ewing's Sarcoma Study (IESS-I) of primarily pediatric patients with localized disease, prophylactic WLI with VAC chemotherapy (vincristine, doxorubicin, and cyclophosphamide) was associated with improved survival and fewer PM relative to those treated with VAC alone [3]. Similarly, other retrospective studies have suggested benefits in disease control and survival with the use of WLI for patients with metastatic ES and clinically evident PM [4–8]. Fractionated WLI at doses between 12 and 21 Gy appears to be well tolerated with acceptable rates of toxicity [3–7, 9–11], although data across all age ranges are lacking. Despite the lack of prospective randomized studies, the available data have supported use of WLI as a component of curative therapy for the majority of pediatric ES patients with PM, and this practice is strongly supported by the U.S. National Cancer Institute (NCI) [12].

However, given the limited efficacy and toxicity data pertaining to WLI for adults, the use of WLI in adult ES patients is appropriately more controversial. Like other sarcomas that impact both adult and pediatric patients, ES presents distinct challenges with respect to forming consensus on best practice treatment strategies across the age spectrum. Clinical studies are ongoing, such as the Euro-Ewing-Intergroup EE99 (COG-AEWSO331) trial, which randomizes patients with pulmonary metastatic disease alone to standard consolidation chemotherapy with WLI or to high dose chemotherapy plus peripheral blood stem cell (PBSC) rescue in patients up to 50 years old. In the absence of robust data regarding efficacy and toxicity, there is no clear consensus on the use of WLI for adult ES patients with PM. In this context, we conducted a survey of expert sarcoma-dedicated oncologists to ascertain practice patterns regarding WLI for adults with ES and PM.

## 2. Methods

We developed a brief survey, in which we described the following clinical scenario:* An adult with excellent performance status presents with a primary ES in the leg and multiple PM. The patient achieves a complete radiographic response after chemotherapy and resection of the primary.* Participants were asked:* Would you give bilateral WLI to (1) this adult patient?, (2) this patient if 20 years old?, (3) this patient if 45 years old?, or (4) this patient if 60 years old? * The survey was emailed to 54 expert sarcoma-dedicated medical and radiation oncologists from adult and pediatric practices located in the US, Europe, and Australia. Experts were identified on the basis of their active involvement in sarcoma clinical trial groups such as EORTC, Children's Oncology Group (COG), NCI Canada Clinical Trials Group, Sarcoma Alliance for Research through Collaboration (SARC), and NRG Oncology. This study was approved by the Dana-Farber/Harvard Cancer Center Institutional Review Board.

Basic demographic information about survey participants was collected including practice discipline, country of practice, adult versus pediatric specialty, and institution. Responses among participant groups were compared using Fisher's Exact Test.

## 3. Results

Thirty-eight of the 54 (70%) emailed participants responded to our survey, including 26 medical and 12 radiation oncologists; 24 adult, 1 adolescent and young adult (AYA), and 13 pediatric oncologists; and 1 from Australia, 14 from Europe, and 23 from the US. Overall, 63% of participants opted to offer WLI to the adult patient, 63% to the 20-year-old, 62% to the 45-year-old, and 50% to the 60-year-old (Table 1).

Table 1 also shows rates of WLI recommendation according to type of oncologist and geographic region. Pediatric oncologists were significantly more likely than adult oncologists to endorse WLI for the adult with no age specified (92% versus 48%, *P* = 0.01), for the 20-year-old (93% versus 46%, *P* = 0.005), and for the 45-year-old (92% versus 46%, *P* = 0.01); the trend was similar for the 60-year-old, but the results were not statistically different (75% versus 38%, *P* = 0.08). No statistically significant differences were seen between responses for medical and radiation oncologists or between oncologists in Europe or Australia versus in the US. Among all categories, WLI was recommended the least for the 60-year-old, but still approximately 50% of the time.

## 4. Discussion

Despite a relatively poor prognosis among patients with metastatic ES, some of these patients are potentially curable, and as such, aggressive multimodal therapy is a standard approach for metastatic ES. Since the 1970s, WLI has been a component of this aggressive therapy [13] although clear data supporting the necessity of its use, particularly for adults, are lacking.

To illustrate practice patterns and to gather expert opinions on this topic, we surveyed a global sample of oncologists with dedicated expertise in sarcoma management on the use of WLI in adults with ES. Almost two-thirds of sarcoma experts in our survey supported WLI for adults with ES and PM up to age 45, and half supported it up to age 60. Although there were no significant differences in recommendations between medical and radiation oncologists, or by practice location, we found that pediatric oncologists were the most likely to recommend WLI for adults at nearly double the rate across all age groups.

In the pediatric literature, there is substantial evidence to support WLI among ES patients with localized disease [3] as well as for those with metastatic disease limited to the lungs in combination with older chemotherapy regimens [4–6, 8]. Data addressing the potential benefits of WLI in combination with more modern dose- and schedule-intensive chemotherapy regimens are lacking, although the ongoing Euro-Ewing-Intergroup EE99 (COG-AEWSO331) trial is asking this important question. Moreover, although many of these early landmark studies did include adult patients, the vast majority of studied patients were young with median ages of 13 (Nesbit et al. [3]), 13.8 (range: 4.6–21.3; Spunt et al. [6]), and 15 (range: 2–45; Paulussen et al. [4]) further highlighting the limited evidence to support or refute the use of WLI in older age groups. Lastly, the appropriate clinical setting in which to employ WLI has never been defined. WLI could be considered (1) as prophylaxis in the setting of local disease, (2) as treatment in the setting of isolated PM either in complete response or present as residual disease following chemotherapy, and (3) as treatment in the setting of PM and extrapulmonary metastases with PM either in complete response or present as residual disease following chemotherapy. At present, the use of WLI is an integral component of many treatment regimens for pediatric patients with ES and PM, but this practice is less standardized for adults in similar clinical situations.

Broad recommendations for the use of WLI in all age ranges of patients with PM are further complicated by the unique side effect profile and comorbidities relevant to each age group. Toxicity concerns in the pediatric population center on the risks of late pulmonary fibrosis, impaired pulmonary function, chest wall hypoplasia/deformity, and secondary malignancy [14]. The latter two are less relevant for adult patients who may be inherently less susceptible to these events, although second malignancies remain an important concern for young adults [15]. Older adults may be more at risk for acute toxicities like pneumonitis or another pulmonary toxicity which is additive to coexisting health conditions and habits such as lung disease and smoking. Significant cardiac toxicity from WLI* per se* has not been described for any age group, but there is a preponderance of evidence associating RT with the heart with subsequent adverse cardiac events for both pediatric and adult cohorts [16–20]. For this reason, potential cardiac toxicity from WLI must be acknowledged, along with consideration of research and efforts to minimize cardiac dose such as the cardiac-sparing IMRT technique for WLI [21].

To help fill the data void relating to WLI for adults, investigators from Memorial Sloan Kettering Cancer Center studied a series of 26 adult patients with ES and PM who were treated with WLI [11]. In this cohort, the median age was 26 years old, and the range was 18–40 years old. Reported 3-year freedom from pulmonary relapse, event-free survival, and overall survival rates were 45%, 38%, and 45%, respectively, and there was excellent treatment tolerability. Acute toxicity was limited to 12 of 26 patients (46%) who experienced Grade 1 toxicities and three patients (12%) who experienced Grade 2 toxicities including esophagitis, fatigue, and nausea. Two patients (8%) developed herpes zoster in a dermatome corresponding to the RT field within three months of RT. There were no observed cases of late pneumonitis, cardiac toxicities, radiographic sequelae, or other toxicities ≥grade 2. Compared with pulmonary metastases only, the presence of extrapulmonary metastases at diagnosis was associated with inferior 3-year PM-free survival (24%), event-free survival (14%), and overall survival (13%); the authors suggested that WLI may not be sufficiently beneficial to justify its use in patients with extrapulmonary metastases. Lower WLI doses of <15 Gy versus ≥15 Gy (*P* = 0.05) predicted for inferior overall survival, whereas a history of smoking predicted for poorer event-free survival (*P* = 0.04) and showed a trend for inferior overall survival (*P* = 0.06). Smokers and former smokers also appeared to have higher rates of acute toxicity (73% versus 36%), but this did not reach significance (*P* = 0.11). No significant differences in outcomes were seen according to response of PM to chemotherapy. This study is an important step towards understanding outcomes of WLI in adult patients with ES but is limited by the relatively young age of patients included and likely selection bias acknowledged by the authors related to the retrospective design, high rate of patients with isolated pulmonary metastases (65%), and high proportion of adults who did not receive WLI at their institution (35%).

In our survey, we identified significant differences in recommendations for WLI between pediatric and adults oncologists. These divergent practice patterns illustrate not only the challenges in the interpretation of limited data sets in rare tumors but also some of the inherent biases that may arise through a practice devoted to either adult or pediatric patients. Practice variation across adult and pediatric providers has been reported in other soft tissue cancers as well. For example, a survey of adult and pediatric oncologists on the treatment of adult medulloblastoma patients similarly demonstrated substantial variation in practice, particularly with the choice of chemotherapeutic agents, likely reflecting the limited data available relevant to older adult population [22]. Likewise, population studies have demonstrated less aggressive protocols for diagnosis, staging, and treatment for adults with Wilms' Tumor relative to their pediatric counterparts and cited this less aggressive approach as an explanation for inferior survival outcomes among adults [23].

Together, these data call for renewed and continued collaboration across the artifactual divides of adult and pediatric oncology to define evidence-based treatment strategies and appropriate prospective trials across the age spectrum. A vibrant form of this collaboration is seen in the emergence of interest in adolescent and young adult (AYA) oncology as a resource-dedicated discipline dedicated to bridging service and survival gaps in the young adult population and in cancers affecting many age groups which span the traditional “lines” demarcating pediatric and adult oncology [24–26]. Efforts of the AYA Oncology Progress Review Group and others have produced an increased understanding of the distinct survival outcomes, survivorship concerns, barriers to care, and challenges to clinical trial enrollment relevant to these patients [26–28]. Increased participation by AYA providers in both patient care and in clinical trial development will undoubtedly expand our understanding of these understudied populations and encourage inclusion of these patients in forthcoming clinical studies.

The ongoing Euro-Ewing-Intergroup EE99 (COG-AEWSO331) trial comparing standard consolidation chemotherapy with WLI to high dose chemotherapy plus peripheral blood stem cell (PBSC) rescue in patients up to 50 years old with EW and PM is one example of such interdisciplinary collaboration, which promises to help elucidate the role of WLI across a broad range of ages. As we await those results, our survey suggests that the majority of expert oncologists would offer WLI for most adults with ES and PM in complete response, particularly for younger patients up to the age of 45. While it may be reasonable to treat younger adults with WLI, outside of a clinical trial, we would recommend caution in older adults with comorbidities due to limited safety data in this patient population. For clinical scenarios of localized disease, PM that do not completely respond to induction therapy, or PM in combination with extrapulmonary disease, we agree with others that the potential benefits of WLI are limited and we would not endorse the use of WLI in these scenarios.

## 5. Conclusion

In this survey of worldwide expert sarcoma-oncologists, approximately two-thirds of respondents supported WLI for adult patients with metastatic ES up to 45 years old, and half supported WLI for patients 60 years old. Pediatric oncologists were most likely to recommend WLI at nearly double the rate across all age groups. These data call for continued collaboration across adult and pediatric oncology to define evidence-based treatment strategies and appropriate prospective trials across the age spectrum. As we await the results of the ongoing Euro-Ewing-Intergroup EE99 study investigating the role of WLI, these data suggest that the majority of expert oncologists would offer WLI for most adults with ES and PM, particularly for younger patients up to the age of 45.

## Figures and Tables

**Table 1:** Surveyed expert oncologists' recommendations for whole lung irradiation for Ewing Sarcoma patients with pulmonary metastases in complete response following chemotherapy.

Whole lung irradiation recommendation	*N*	Adult^*∗*^	*P*	20-year-old	*P*	45-year-old	*P *	60-year-old	*P*
All oncologists surveyed	38	63%		63%		62%		50%	

*Oncologist discipline *									
Medical oncologist	26	58%	NS	58%	NS	60%	NS	46%	NS
Radiation oncologist	12	73%		75%		67%		58%	
AYA/pediatric oncologist	14	92%	0.01	93%	0.005	92%	0.01	75%	0.08
Adult oncologist	24	48%		46%		46%		38%	

*Oncologist country *									
Europe/Australia	15	57%	NS	60%	NS	53%	NS	40%	NS
United States	23	67%		65%		68%		57%	

^*∗*^Adult age not specified.

*P* = 2-tail Fisher Exact Test *P* value; NS = not significant; AYA = adolescent and young adult.
